# Neurolisteriosis in a previously asymptomatic patient with serum IgM deficiency: a case report

**DOI:** 10.1186/s12883-020-01900-3

**Published:** 2020-08-31

**Authors:** Kostas Patas, Theodoros Mavridis, Katerina Psarra, Vassilis E. Papadopoulos, Georgia Mandilara, Alexandra Tsirogianni, Sophia Vassilopoulou, Stylianos Chatzipanagiotou

**Affiliations:** 1 Department of Medical Biopathology, Eginition Hospital, National and Kapodistrian University of Athens, Medical School, Athens, Greece; 2 Department of Neurology, Eginition Hospital, National and Kapodistrian University of Athens, Medical School, Athens, Greece; 3 Department of Immunology and Histocompatibility, Evangelismos General Hospital, Athens, Greece; 4 National School of Public Health & Central Public Health Laboratory, Hellenic Centre of Disease Control and Prevention, Vari, Greece; 5 Department of Clinical Microbiology and Medical Biopathology, National and Kapodistrian University of Athens, Aeginition Hospital, Ave. Vassilissis Sophias 72-74, 115 28 Athens, Greece

**Keywords:** Neurolisteriosis, *Listeria monocytogenes*, IgM deficiency

## Abstract

**Background:**

*Listeria monocytogenes* is an opportunistic pathogen of the central nervous system commonly associated with impaired cell-mediated immunity. We hereby present a case of adult neurolisteriosis where the only immunological feature persistently present was serum IgM deficiency, suggesting that non-specific humoral immunity may also play a central role in the control of neuroinvasion by *Listeria monocytogenes*.

**Case presentation:**

A 62-year-old male who had never experienced severe infections presented with headache, nuchal rigidity and confusion. Neuroimaging was normal and lumbar puncture revealed pleiocytosis (760 leukocytes/mm^3^) and hypoglycorrhachia (34 mg/dL). The patient was treated empirically for bacterial meningitis. Indeed, further analysis of the CSF showed infection by *Listeria monocytogenes*, which was accompanied by reduced serum IgM levels that persisted well beyond the period of acute bacterial infection. Levels of IgG and IgA isotypes, along with peripheral blood counts of major leukocyte subsets, were at the same time largely preserved. Intriguingly, the absence of membrane-bound IgM on B cells was essentially complete in the acute post-infection period leading to a remarkable recovery after 12 months, suggesting that mechanisms other than defective membrane expression are underlying serum deficiency.

**Conclusions:**

As far as we know, this is the first reported case of neurolisteriosis associated with IgM deficiency in an adult individual without a history of severe infections or other underlying conditions. A possible role of circulating IgM against invasive disease caused by *Listeria monocytogenes*, particularly in the early course of host-pathogen interaction, is discussed.

## Background

*Listeria monocytogenes* (*Lm*), a Gram-positive facultative intracellular bacterium, is an uncommon, highly opportunistic human pathogen. The natural route of infection is through the gastrointestinal tract following consumption of contaminated food. After ingestion, *Lm* crosses the intestinal-blood barrier aided by active endocytosis by epithelial cells [1] and disseminates through the enteric lymphatics to the primary target organs, i.e. liver and spleen [2]. In immunocompetent adults, this infectious process remains largely subclinical as the pathogen is being efficiently cleared by cell-mediated immunity [3]. Exposure to higher infective doses usually results in self-limited febrile gastroenteritis.

However, certain groups characterized by impaired cellular immune responses, i.e. pregnant women and those with immunosuppressive comorbidities, are at an increased risk for invasive infection by *Lm* [4]. In these populations, an inadequate resolution of primary infection may escalate into febrile bacteremia and eventually secondary invasive disease [5]. In particular, *Lm* is notorious for its tropism towards two immunologically “privileged” sites: the fetoplacental unit in pregnant women [6] and the central nervous system (CNS) in otherwise immunocompromised individuals [7].

We hereby present a case of CNS infection caused by *Lm* (neurolisteriosis) in a previously asymptomatic adult patient. The only immunological feature repeatedly present in this patient – both throughout and following the infection – was serum IgM deficiency, suggesting that this non-specific humoral factor may play an early central role in the control of neuroinvasion by *Lm*.

## Case presentation

A 62-year-old male patient was admitted to the emergency department of our hospital with fever, confusion, irritation and severe bitemporal headache with occipital radiation. The symptoms had exacerbated acutely 1.5 h prior to hospital arrival (estimated NPRS: 9/10) and awakened the patient from sleep. According to his relatives, the patient also had fever (up to 39.5 °C), headache (estimated NPRS: 3/10) and bouts of vomiting during the last 3 days before admission. From his past medical history, the patient had arterial hypertension treated with irbesartan and atenolol. His personal and family history was free of neurological diseases and severe infections.

Vital signs upon admission were as follows: blood pressure 125/80 mmHg, heart rate 76 bpm, body temperature 38.8 °C and oxygen saturation 94% on room air. The basic neurological examination revealed an alert (GCS: 14/15, E:4/4, V:5/5, M:5/6) but disoriented and confused patient with nuchal rigidity and opisthotonus. There were no focal neurological deficits from the cranial nerves. Upper and lower limb strength appeared normal. Examination of the sensory and cerebellar function as well as systemic examination were not possible due to limited cooperation.

A chest X-ray was normal and an ECG showed sinus rhythm. Computed tomography of the brain was negative for acute intracranial pathology. In specific, no mass, haemorrhage or hydrocephalus was identified. Basal ganglia and posterior fossa structures were normal. No established major vessel vascular territory infarct and no intra or extra axial collection were reported. The basal cisterns and foramen magnum were patent. The air cells of the petrous temporal bone were non-opacified and no fracture was demonstrated.

Initial laboratory workup demonstrated a white blood cell count of 16.3 × 10^3^/uL (88% polymorphonuclear cells; normal ranges: 4.0–9.0 × 10^3^/uL and 42–85%, respectively), hemoglobin 18.5 g/dL (normal range: 13.5–18.0 g/dL) and platelets 192 × 10^3^/uL (normal range: 150–400 × 10^3^/uL). Blood biochemistry and coagulation were within reference limits except for high glucose (202 mg/dL; normal range: 70–115 mg/dL), LDH (534 U/L; normal range: 170–480 U/L), γ-GT (84 U/L; normal range: 10–49 U/L) and C-reactive protein (8.9 mg/dL; normal range: < 0.5 mg/dL). Lumbar puncture was performed while the patient was in the emergency department. Cerebrospinal fluid (CSF) assessment revealed a turbid fluid containing 760 leukocytes/mm^3^ with polymorphonuclear predominance (85%), markedly elevated protein (302 mg/dL; age-adjusted normal range: 15–60 mg/dL) and low glucose (34 mg/dL; normal range: 40–70 mg/dL). A remarkably increased CSF/serum albumin index (50.49; age-adjusted normal range: < 8.13) indicated significant dysfunction of the blood-brain barrier, with no signs of local IgG production (CSF IgG index: 0.46; normal range: < 0.65).

Despite a negative Gram stain of the CSF, an initial impression of bacterial meningoencephalitis was rendered and the patient was put on empirical intravenous therapy with ceftriaxone (2 g bid), vancomycin (1 g bid), ampicillin (2 g every 4 h) and dexamethasone (8 mg every 6 h). Blood and CSF cultures were obtained before treatment initiation and screening for common viral and bacterial pathogens involved in human CNS infection was carried out in CSF using a multiplex PCR assay (MeningoFinder 2SMART, PathoFinder; Maastricht, The Netherlands). While waiting for culture results, a positive PCR reaction for *Lm* was obtained and dexamethasone was discontinued, as it is contraindicated in patients with neurolisteriosis [8]. Of note, co-detection of herpes simplex virus (HSV) type 1 DNA by the PCR assay was not taken into further consideration (see Discussion in the following section).

By day 3, the patient was already showing signs of clinical improvement, i.e. he was apyretic, GCS: 15/15, fully oriented in place, time, self and event. There was no nuchal rigidity and headache attacks were milder and infrequent (NPRS: 4/10) and were relieved with intravenous paracetamol. Brain MRI showed no abnormal focal areas of altered signal intensity in the temporal lobes or the cerebral hemispheres, brainstem and cerebellum. Appearance and intensity of brain parenchyma was normal. The ventricular system and cisternal spaces appeared normal as well. There was no evidence of intracranial space occupying lesion or obvious vascular anomaly and no shift of the midline structures was observed. An EEG was also performed and showed no abnormalities. In specific, the background waking state activity consisted of well-regulated medium amplitude alpha activity at 11 Hz symmetrically in the posterior head regions and attenuated with eye opening. Hyperventilation did not reveal any abnormal phenomena.

The CSF culture on blood agar grew a Gram-positive and catalase-positive coccobacillus which appeared as rod following enrichment in thioglycolate broth. The culture isolate was identified as *Lm* by means of the MicroScan Gram-positive panel (Biotype 306,403; Beckman Coulter; Atlanta, Georgia, USA) as well as by reverse dot blot hybridization (Bacterial CNS Flow Chip, Master Diagnostica; Granada, Spain). In keeping with this identification, the patient recalled upon specific questioning a recent consumption of ready-to-eat dairy products. Serotyping by PCR according to Doumith et al. [9] placed the *Lm* isolate into serovar group 1/2a, 3a. No *Lm* was recovered from blood cultures.

Antibiotic susceptibility test of the CSF isolate was performed according to the Clinical and Laboratory Standards Institute criteria via standard disk diffusion method on blood agar. The isolate was found susceptible to ampicillin, vancomycin, ciprofloxacin and aminoglycosides (amikacin, gentamicin, tobramycin) but resistant to cephalosporins (cefuroxime, ceftazidime, cefepime). Ceftriaxone was therefore discontinued, while vancomycin and ampicillin were continued for a total of 2 and 3 weeks, respectively.

A second lumbar puncture on day 10 showed a clear amelioration of CSF parameters paralleling clinical improvement: 59 leukocytes/mm^3^ with lymphocytic predominance (85%), slightly elevated protein (88 mg/dL) and normalized glucose (54 mg/dL). CSF culture and PCR were both negative. By day 14, the patient was afebrile, fully oriented and free of headache. He was discharged in good clinical condition upon completion of treatment with ampicillin.

In view of the high postprandial glucose levels upon admission, we first ruled out diabetes mellitus on the basis of normal HbA1c levels (5.2%; normal range: 4.0–6.0%). In an effort to investigate further the presence of immunosuppressive comorbidities, we screened for both humoral and cellular immune abnormalities. Routine serology revealed persistently low serum IgM levels as measured by nephelometry, both during hospitalization as well as at 3- and 12-month follow-ups (see Table 1).

**Table 1:** Serum levels of immunoglobulins

	Day 3	Day 18	Month 3	Month 12
IgG (700–1600 mg/dL)	770	*689*	955	1070
IgA (70–400 mg/dL)	182	220	202	250
IgM (40–230 mg/dL)	*< 16.8*	*23*	*20.7*	*21.5*

*Reference ranges are given in parentheses*

By contrast, serum levels of IgG and IgA were largely normal. IgG subclasses at 12-month follow-up were also found within reference limits and repeated immunoelectrophoreses showed no abnormality. In addition, the patient was HIV-seronegative, with normal levels of complement components C3 and C4 and negative antinuclear antibody titers (< 1:80).

Whole-blood immunophenotyping by flow cytometry (Navios EX, Beckman Coulter) took place on the last day of hospitalization (day 21 – i.e., 18 days after dexamethasone discontinuation) as well as at 12-month follow-up. Although absolute counts of CD19+ B cells were normal on both occasions, surface expression of IgM was virtually absent (0.16%) in month 1, while it substantially recovered (77.58%) on total B cells after 12 months. The majority of B cells expressing membrane-bound IgM at 12-month follow-up were CD27-IgD+ naïve B cells, notwithstanding the fact that this subset was IgM-negative at baseline (+ 66.86% recovery). Small recoveries of IgM expression (ca. + 5%) were nevertheless observed on both non-isotype-switched and isotype-switched CD27+ memory B cells as well (see Table 2). Of note, we did not observe major abnormalities in other leukocyte subsets (granulocytes, monocytes, T cells and NK cells), while modest increases of the CD4+/CD8+ T cell ratio and γδ T cells were confined to the acute post-infection period.

**Table 2:** Absolute counts of major leukocyte subsets and frequencies of B cell subsets

	Month 1 (cells/uL)		Month 12 (cells/uL)	
Granulocytes (1500–6600/uL)	5100		4700	
Monocytes (< 900/uL)	600		500	
Lymphocytes (1500–3500/uL)	2800		2400	
CD3+ T cells (1500–1900/uL)	1820		1982	
CD3 + CD4+ T cells (663–1477/uL)	1322		1426	
CD3 + CD8+ T cells (342–754/uL)	*340*		476	
RATIO CD4+/CD8+ (1.4–2.6)	*3.9*		*3.0*	
CD3 + TCRγδ+ (27–179/uL)	*196*		94	
CD3-CD16/56+ NK cells (100–350/uL)	269		*408*	
CD19+ B cells (150–400/uL)	186		209	
	Month 1 (% of B cells)		Month 12 (% of B cells)	
	Total	IgM+	Total	IgM+
CD27-IgD+ naïve B cells	82.59	none	74.11	66.86
CD27 + IgD+ non-switched memory B cells	2.75	0.16	7.14	5.25
CD27 + IgD- switched memory B cells	10.84	none	15.18	5.29

*Reference ranges are given in parentheses*

## Discussion and conclusions

IgM antibodies constitute a class of multifunctional evolutionary conserved antibodies which are the first to be produced during a primary immune response. They play a central role in the development of early humoral immunity to invading pathogens as well as the maintenance of immune and tissue homeostasis. As a result, patients with IgM deficiency, when not asymptomatic, may present with a wide range of clinical manifestations, including invasive or recurrent infections, atopy, autoimmunity or even malignancy [10].

According to the European Society for Immunodeficiencies, the following clinical criteria should be met for a diagnosis of selective IgM deficiency: infections (either invasive or recurrent, usually bacterial), low IgM serum/plasma levels (with normal levels of IgA, IgG and IgG subclasses), normal IgG antibody response to all vaccinations and exclusion of T cell defects (ESID Registry, www.esid.org). As we were not able to assess antibody response to vaccinations in our patient (he refused any type of vaccination after his recovery), nor did we perform any functional analysis, we refrained from using the term “selective” [11].

Our patient denied a history of recurrent infections, autoimmune disease, malignancy, diabetes, alcoholism and use of immunosuppressive medication. Similarly, thorough clinical examination and targeted paraclinical assessment did not reveal any underlying medical comorbidities. This is in agreement with a previous review reporting that 36% of the adult patients presenting with neurolisteriosis did not have a recognizable immunosuppressive comorbidity [12]. In addition, and to the best of our knowledge, no IgM levels were assessed in any other clinical setting prior to this infection in our patient. However, a chronic history of allergic rhinitis was disclosed upon more targeted history questions, which is well in line with observations that atopic manifestations are common in IgM-deficient patients [10, 13].

CNS infections caused by *Lm* differ from those brought about by other bacteria in that they may have a sub-acute course, i.e. usually longer than 24 h before admission [7]. This is consistent with a history of antecedent fever, headache and bouts of vomiting in our patient during the last 3 days before onset, suggesting efficient bacterial translocation across the intestinal-lymphatic barrier prior to dissemination into the CNS [2]. In concordance also with previous reports on the low yield of the Gram stain in neurolisteriosis [12], a negative result was obtained from direct examination of the CSF. This probably results from a quantitatively modest dissemination of *Lm* in the CSF, secondary to the tropism this pathogen demonstrates for the brain parenchyma [7].

Of note, our PCR assay showed co-detection of HSV-1 in CSF, this finding was however not in keeping with negative findings on brain MRI and EEG as well as the patient’s overall clinical improvement in the absence of antiherpetic therapy. Given that the patient was HSV-1 IgG-seropositive, we presume that co-amplification of the HSV-1-associated target gene resulted from bystander reactivation of the virus initiated by adjunctive dexamethasone and/or the sympathetic response to the bacterial infection [14]. Secondary reactivation of Herpesviruses in response to disease stress and local inflammation has been previously inferred in some cases of CNS infections [15, 16].

The underlying pathophysiological mechanisms of IgM deficiency are far from clarified [10]. As such, we aimed first to screen for B cell subset deviations relevant to this immunoglobulin isotype. Expression of membrane-bound IgM in our patient was virtually absent during the first measurement (month 1; acute post-infection). Reduced expression of surface IgM on total B cells has been described in a small number of patients characterized by decreased serum IgM [17, 18], but the majority of adult patients seems to have normal surface expression [10, 13]. Given that the first measurement took place on the last day of hospitalization, possible effects of treatment on membrane-bound IgM expression cannot be ruled out in our case. Regarding secreted IgM however, any lasting effects of dexamethasone are rather unlikely, as serum levels of this isotype do not seem to be affected by systemic glucocorticoid therapy (reviewed in [19]).

Intriguingly, we observed a marked recovery of surface IgM expression on B cells after 1 year (month 12; infection-free), which did not coincide with restored serum levels. Recovery of surface expression occurred to a percentage (77.58%) comparable to that seen on total B cells of previously described IgM-deficient patients [13], suggesting that mechanisms other than defective membrane expression might be at play. Of note, recovery of surface IgM was most markedly seen on naïve as opposed to memory B cells, which is in accordance with a recent study showing reduced formation of IgM-expressing memory B cells in IgM-deficient patients [20].

Along the same line, we noticed that the frequency of CD27 + IgD + IgM+ B cells was both in month 1 and month 12 measurements (Table 2) lower than the expected mean (i.e., 15%) based on previous measurements in the peripheral blood of healthy adult donors [21]. These cells have a surface phenotype of splenic marginal zone B cells and decreased percentages have already been observed in some adult IgM-deficient patients [20], but not in others [22]. Importantly, marginal zone B cells constitute one of the very first cell types that come into contact with blood-borne pathogens, including *Lm*, as well as a major source of rapidly produced IgM during experimental bacteremia [23, 24]. Therefore, this B cell subset might be of pathophysiological relevance to our case.

Resistance of *Lm* to phagocytosis is mediated by the ability of the microorganism to escape from the vacuole, grow and move within the cytosol and achieve actin-based intercellular spreading [2]. Such largely intracellular physiology conceivably provides little opportunity for antigen encounter by B cells. Indeed, resistance to *Lm* infection is predominantly cell-mediated, as exemplified by seminal studies showing that immunity can be transferred by sensitized mononuclear cells but not by antibodies [25]. However, under certain experimental conditions, IgM has been shown to play a crucial part in early anti-listeria responses. For instance, by comparing the opsonic activity of human adult and neonatal cord blood sera, Bortolussi et al. have shown that, in the presence of complement, optimal opsonization of *Lm* requires the IgM (but not the IgG) fraction of serum [26]. Of further relevance to our case, preclinical approaches employing B cell- and IgM-deficient mice have also demonstrated a decisive involvement of the IgM class in preventing early *Lm* dissemination to vital tissues, including the CNS [27].

As far as we know, this is the first reported case of neurolisteriosis associated with IgM deficiency in an adult individual without a history of severe infections or other underlying conditions. Serum IgM deficiency was serendipitously found during hospitalization and persisted well after recovery, which led us to presume that this is an underlying immunological defect possibly relevant to the infection by *Lm*. Therefore, opportunistic infections such as listeriosis, in clinically healthy individuals, warrant thorough immunological examination in order to disclose possible predisposing factors.

## Data Availability

Not applicable.

## Abbreviations

CNS: Central nervous system
CSF: Cerebrospinal fluid
ECG: Electrocardiogram
EEG: Electroencephalogram
GCS: Glasgow coma scale
HSV: Herpes simplex virus
*Lm*: *Listeria monocytogenes*
MRI: Magnetic resonance imaging
NPRS: Numeric pain rating scale
PCR: Polymerase chain reaction
